# Effects of insufficient serine on health and selenoprotein expression in rats and their offspring

**DOI:** 10.3389/fnut.2022.1012362

**Published:** 2022-09-14

**Authors:** Yiqun Liu, Jianrong Wang, Qin Wang, Feng Han, Lili Shi, Chao Han, Zhenwu Huang, Liang Xu

**Affiliations:** ^1^Department of Nutrition and Metabolism, National Institute for Nutrition and Health, Chinese Center for Disease Control and Prevention, Beijing, China; ^2^The Key Laboratory of Micronutrients Nutrition, National Health Commission of The People's Republic of China, Beijing, China; ^3^Urology Department, The Third Medical Centre of Chinese PLA General Hospital, Beijing, China

**Keywords:** serine, PHGDH inhibitor, GPx, SELENOP, DIO2

## Abstract

**Objective:**

To observe the impact of insufficient exogenous and/or endogenous serine on selenoprotein expression and health of pregnant rats and their offspring.

**Method:**

Experiment 1 was conducted in male rats, in which the dose-dependent effects of serine on selenoprotein expression and thyroid hormones (T3, T4 and TSH) were investigated by feeding either a serine adequate diet (20C), serine-deprived diet (20CSD) or 20CSD with different serine levels (0.5, 1.0, and 2.0 times the amount of serine in 20C). In experiment 2, a PHGDH inhibitor was administrated to pregnant rats fed either 20C or 20CSD. Blood and organ tissues of pregnant rats and offspring were subjected to the analyses of thyroid hormone, serine and homocysteine and GPx3 and SELENOP in plasma and expression of GPx1 and DIO1, 2 in tissues respectively.

**Result:**

In experiment 1, plasma SELENOP and GPx3 levels in adult male rats increased with the increasing dose of serine. Immunohistochemical results showed that GPx1 expression in liver and kidney of male rats also increased with increasing serine supplementation. Amongst all diet groups, only male rats fed 20CSD had significantly lower plasma TSH and T4 levels (*P* < 0.05). In experiment 2, GPx1 and DIO2 expression in the liver and kidney were suppressed in pregnant rats administered with a PHGDH compared to those who were not (*P* < 0.05). There were no significant differences in plasma T4 and T3 amongst all diet groups (*P* > 0.05). Also, offspring born to pregnant rats administered with a PHGDH inhibitor exhibited slower growth rates and hyperhomocysteinemia compared to offspring from mothers not administered with the inhibitor (*P* < 0.05). Conclusions: Insufficient exogenous serine through the diet decreased selenoprotein synthesis in adult male rats. However, this was not observed in pregnant rats, whereby exogenous or endogenous serine deficiency had no effect on the selenoprotein levels. A possible explanation is that dams may have an adaptive mechanism to limit maternal serine utilization and ensure adequate supply to the fetus.

## Introduction

**Figure 1:**
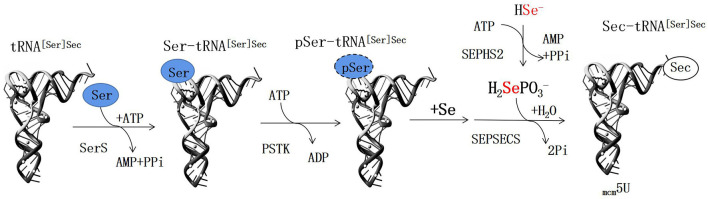
This schematic illustrates the *de novo* biosynthesis of Sec-tRNA^[Ser]Sec^.

Selenium is an essential trace element and it plays a role in many biological functions as selenoproteins, which contain the amino acid selenocysteine (Sec). Sec-transfer RNA (tRNA), a specific tRNA for selenocysteine, is often transformed from Ser-tRNA [a tRNA specific for serine (Ser)] during the de novo synthesis of Sec-tRNA^[Ser]Sec^ and not directly aminoacylated with Sec (1) (Figure 1). In other words, serine is essential for the expression of selenoproteins including deiodinases.

It was reported that the addition of serine to conventional medium can greatly promote the synthesis of selenoprotein (SELENOP) and glutathione peroxidase 1, (GPx1) in HepG2 cells (2). *In vivo*, serine supplementation greatly increased selenoproteintranscription and Se-containing enzyme (GPxs) activity in organs and tissues (such as skeletal, muscle and liver) of pigs (3). Also, the addition of serine in the maternal diet during the third trimester and subsequent lactation resulted in a greatly improved Se nutritional status in both sows and their offspring, in which may result in higher body weight offsprings (4). We have recently reported that the total amount of serine from dietary protein intake daily was associated with the nutritional status of Se in Chinese lactating women living in the traditional endemic areas of Keshan diseases (5).

Serine is known as a nonessential amino acid. Mammals can adaptively mobilize the synthesis of endogenous serine when dietary serine is insufficient. There are two pathways for the biosynthesis of endogenous serine, one is the de novo synthesis pathway of serine (SSP) derived from glycolysis bypass (6, 7), and the other is the mutual conversion of glycine through serine hydroxymethyltransferase (SHMT) enzymatic synthesis (8). Thus, mutations in the genes of enzymes participating in the de novo biosynthesis of serine including 3-phosphoglycerate dehydrogenase (PHGDH), phosphoserine aminotransferase 1 (PSAT1), and phosphoserine phosphatase (PSPH), coupled by systemic or local serine deficiency, can cause a broad spectrum of phenotypic disorders (9, 10). The key enzyme of de novo serine synthesis pathway, PHGDH, is high-expressed for endogenous serine synthesis providing enough glycine and one-carbon units for nucleic acids biosynthesis and proteins expression to keep the continuous proliferation of tumor cells. However, there are no studies investigating the expression of selenoproteins including deiodinases which are responsible for thyroid hormones metabolism when serine biosynthesis is impaired.

Here, a well-established animal model of impaired serine biosynthesis induced by an inhibitor of PHGDH (11) was used to observe the negative effects of serine deficiency on deiodinase expression in pregnant rats and development of their offspring.

## Materials and methods

### Chemicals

Serine was purchased from Sigma-Aldrich (St. Louis, Mo). All other chemicals were of analytical grade and purchased from either Wako Pure Chemical (Osaka, Japan) or Hitachi Company (Tokyo, Japan). Dietary ingredients were purchased from Beijing HFK Bioscience Co., LTD, China. NCT-503, a PHGDH inhibitor (MedChem Express) was obtained from Shanghai Haoyuan Chemexpress Co., Ltd (China). Human glutathione peroxidase (GPx) and SELENOP ELISA Kits were from Andy Gene Biotechnology Co., Ltd (Beijing, China).

### Animals and diets

Six-week-old male Wistar rats (weighing 120–140 g) and pregnant rats were purchased from Beijing Vital River Laboratory Animal Technology Co., Ltd. (License No. SCXK (Jing) 2018-0006). They were individually housed in a temperature (23–25°C) and humidity (40%−60%) controlled animal room with a 12-h light-dark cycle (lights on from 07:00 to 19:00 h). All rats were given free access to water and a 20% casein diet during the 5 days acclimatization period. In experiment 1, male rats were fed either a serine sufficient diet (20C) or serine-deprived 20C diet (20CSD) for 60 days. The rats were then administered with either double distilled water in 20C or supplemented with 0.5, 1.0 and 2.0 times the amount of serine in 20C (diluted in distilled water) daily via gavage feeding for the next 30 days. The five experimental groups of Experiment 1 were as follow: (a) 20C + distilled water, (b) 20CSD + distilled water, (c) 20CSD + 0.5 × Ser, (d) 20CSD + 1.0 × Ser and (e) 20CSD + 2.0xSer. It is important to note that both glycine and serine were removed from all 20CSD diets to prevent the mutual conversion of glycine to serine (12, 13). Male rats were fed their allocated diets for 60 days to investigate the dose-dependent effects between serine, selenoprotein and thyroid hormone. Composition of all five diet groups were listed in Table 1. In experiment 2, fourty pregnant rats were randomly assigned to one of the four diet groups: (1) 20C, (2) 20C + NCT-503, (3) 20CSD, (4) 20CSD + NCT-503. Pregnant rats were administered with NCT-503 on the first day of gestation. NCT-503 (PHGDH) was dissolved in a vehicle of 5% ethanol, 35% PEG 300, and 60% of an aqueous 30% hydroxypropyl-β-cyclodextrin solution and injected at 30 mg/kg i.p (11). The dose was adjusted according to the weight of each rat, and the injection volume was 100 μL. Pregnant rats were given free access to the experimental diets and water until weaning of their offspring. Offspring were weighed and measured once a day, their fur, mental state were also observed. At the end of the experiment, the mother rats were euthanized between 9:00 and 11:00 h without prior food deprivation as it was shown that non-fasting pHcy concentration was liable to dietary treatment in humans (14). This study was approved by the Animal Care and Use Committee of National Institute of Nutrition and Health, Chinese Center for Disease Prevention and Control.

**Table 1:** Two groups of feed formula.

**Ingredient (g/kg)**	**20C**	**20CSD**
Casein	200	–
Casein (serine + glyine free)	–	200
Cystine	3	3
Corn starch	397	397
Maltodextrin	132	132
Sucrose	100	100
Cellulose	50	50
Soybean oil	70	70
Choline hydrogen tartrate	2.5	2.5
AIN mineral	35	35
AIN vitamin	10	10

20C, 20% casein diet; 20CSD, serine-deprived 20C.

### Tissue collection and histological examination

Blood plasma was separated from heparinized whole blood by centrifugation at 3,000 × *g* for 10 min at 4°C and was stored at −30°C for further analysis. Whole liver was quickly removed, rinsed in ice-cold saline, blotted with filter paper, cut into two portions, one portion was weighed and frozen in liquid nitrogen. Liver samples and other organs were stored at −80°C until further analysis. Another portion of the liver and organs were weighted and histopathological examination (15, 16). The organs included kidney, pancreas, testis, uterus and brain tissues.

### Blood biochemistry analysis

Total cholesterol (TCHO), triacylglycerol (TG), glucose (Glu), thyroid-stimulating hormone (TSH), Triiodothyronine (T3), thyroxine (T4), and homocysteine (Hcy) levels in plasma were measured using an automatic biochemical analyzer (Hitachi 7600-210E, Tokyo, Japan). Blood serine concentration was measured by an amino acid autoanalyzer. All assays were performed according to the manufacturer's protocols.

### Se detection of whole blood in rats

For the analysis of whole blood Se, 1 g of a previously heated (25°C) and shaken blood samples were weighed into 10 ml Teflon microwave vessels and 2 ml of 65% HNO_3_ was added. During digestion, the samples were digested at 120°C for 10 min, after which the temperature was ramped to 120°C (within 8 min), and then to 160°C for 10 min, and finally to 175°C for 20 min using a CEM MARS Xpress microwave system (CEM, Matthew, NC, USA). The cooled, digested samples were diluted to 10 ml with ultrapure water and analyzed for total Se content by inductively coupled plasma mass spectrometry (ICP-MS).

### Double-antibody sandwich ELISA assay for SELENOP and GPx

After cutting samples, check the weight and add cold PBS (PH7.4), and maintain samples at 2–8°C after melting. The samples were homogenized by hand or grinders and centrifugated for 20 min at the speed of 2,000–3,000 rpm. The supernatant was collected for detection. The double-antibody sandwich ELISA assay for quantification of SELENOP and GPx was used according to the instructions of a validated SELENOP/GPx-specific ELISA kit (2). Setting five standards for drawing calibration curve, the absorbance was measured using a microplate reader at a wavelength of 450 nm.

### Western blot analysis

Liver tissue was broken up with high-speed tissue grinder. Protein Lysates from Tissues were directly lysed in RIPA Lysis Buffer (Strong) containing 50 mM Tris-HCl (pH 7.4), 150 mM NaCl, 0.1% SDS, 1% Triton X-100, 1% Sodium Deoxycholate and 1 mM phenylmethane sulfonate fluoride (PMSF) for 30 min, and disrupted with a sonicator using five 5 s pulses over ice. The homogenate was separated by centrifugation at 12,000 × g for 20 min at 4°C to obtain the supernatant. Protein concentration was measured using the Bradford method with the BCA Protein Assay Kit (Solarbio) to measure protein levels for normalization purposes. Equal amounts of proteins (40 g/lane) were separated by 10% SDS–PAGE (Bio-Rad) and blotted onto a 0.45 μm pore-sized PVDF membrane (Millipore). Membranes were blocked for 10 min at room temperature in NcmBlot Blocking Buffer (NCM Biotech), washed twice in TBST (20 mM Tris, 150 mM NaCl, 0.1%Tween-20) for 20 min at room temperature. Membranes were incubated for 1 h at room temperature, then were incubated overnight at 4°C with DIO1 polyclonal antibody (ThermoFisher,Catalog # PA5-100139), DIO2 polyclonal antibody (Abcam,Catalog # ab77481) using a dilution of 1:3,000 and GAPDH Monoclonal antibody (Proteintech,Catalog # 60004-1-Ig) at dilution of 1:20,000, washed three times with TBST for 30 min. Thereafter, incubated for 1h with SA00001-2 [HRP-conjugated Affinipure Goat Anti-Rabbit IgG(H + L)] or SA00001-1 [HRP-conjugated Affinipure Goat Anti-Mouse IgG(H + L)] as secondary antibody (Proteintech,dilution 1:2,000), then washed three times with TBST for 30 min. Immunoreactive bands were visualized by detection of peroxidase activity by chemiluminescence (Aq Super ECL Reagent, AQ). An Gel Imager (AzureSpot, USA) was used for detection, and quantification of immunoreactive bands was carried out by ImageJ 1.53s 19 software.

### Immunohistochemically analysis

The tissue chip was soaked in xylene for 10 min, replaced the xylene and soaked for another 10 min. Soaked in gradient ethanol (100, 95, 70%) for 5 min each and followed by washing 2–3 times with PBS for 5 min each. 3% H_2_O_2_ (80% methanol) was added dropwise on TMA and left for 10 min at room temperature (RT). Then, the slides was washed 2–3 times with PBS for 5 min each. Added 0.01 M sodium citrate buffer solution (pH 6.0) to boiling water. Covered the stainless steel pressure cooker without locking it. Placed the slides on the metal staining rack and slowly pressurized it to soak the slides in the buffer for 5 min, then locked the lid and the small valve will rise up. After 10 min, removed the heat source, put it in cold water, and open the lid when the small valve sinks. After antigen retrieval, the slides were washed in PBS for 2–3 times for 5 min each (17).

Normal goat serum blocking solution was added dropwise, 37°C for 20 min. Excess liquid was shaken off. Added antibody I 50 μl dropwise, 37°C 1 h (GPx-1:300 dilution). Thereafter washing the slides three times with PBS for 5 min each. Added 40–50 μl of Antibody II dropwise, and incubated the slides at 37°C for 1 h (Secondary antibody: 300-fold dilution), and then washed the slides three times with PBS for 5 min each. DAB color development for 5–10 min, mastering the degree of staining under the microscope. Then the slides was rinsed with tap water for 10 min. Hematoxylin re-stained the slides for 30 s, rinsed after dropping. Rinsed the slides with tap water for 10–15 min. Thereafter by dehydration, transparency, sealing, and microscopy, the immunohistochemistry was performed with grayscale analysis of protein levels using IMAGE PRO PLUS 6.0 after imaging.

### Statistical analysis

All values were expressed as mean ± SEM. Data were analyzed by one-way ANOVA, and differences among the experimental groups were analyzed by the Tukey test when the *F* value was significant. A probability of *P* < 0.05 was considered to be statistically significant. Statistical analysis was performed with SPSS 22.0 software (SPSS Inc., Chicago, IL, USA).

## Results

### Experiment 1 — Conducted in adult male rats

#### Effects of serine on plasma CHO, TG, Hcy, Se, serine and thyroid hormone levels

No significant changes in plasma Se concentration were observed in male rats fed 20CSD regardless of serine supplementation concentration (Table 2). Compared to the 20C group, plasma thyroid-stlmulating hormone (TSH) and thyroxine (T4) levels were significantly lower in the 20CSD group (*P* < 0.05); both were positively associated with serine concentration supplemented in the 20CSD diet. On the other hand, triiodothyronine (T3) was not affected by neither deprivation nor supplementation of serine in male rats (Table 2). Plasma cholesterol (CHO) and triacylglycerol (TG) concentrations in male rats fed 20CSD were significantly lower than those fed 20C (*P* < 0.05); however, serine supplementation had no effect on this decrease (Table 2).

**Table 2:** Effects of supplementation of serine-deprived diet with serine (0.5, 1.0, and 2.0 times the amount of serine in 20C) on plasma Se concentration and other variables in rats fed a 20% casein diet (experiment 1).

**Diet**	**20C**	**20CSD**	**+ 0.5 Ser**	**+ 1.0 Ser**	**+ 2.0 Ser**
TCHO (mmol/L)	1.98 ± 0.05^a^	1.58 ± 0.06^bc^	1.55 ± 0.05^c^	1.72 ± 0.07^b^	1.63 ± 0.05^b^
TG (mmol/L)	1.51 ± 0.21^a^	0.82 ± 0.18^b^	0.75 ± 0.06^b^	0.95 ± 0.08^b^	1.00 ± 0.18^b^
TSH (μlU/L)	1,136 ± 25^a^	857 ± 24^d^	1,024 ± 24^b^	921 ± 25^cd^	956 ± 26^bc^
T3 (pmol/L)	32.8 ± 0.7^a^	30.9 ± 0.89^a^	32.0 ± 0.94^a^	27.1 ± 0.58^b^	31.1 ± 0.82^a^
T4 (pmol/L)	1,243 ± 21^ab^	1,077 ± 25^d^	1,184 ± 23^bc^	1,112 ± 42^cd^	1,314 ± 18^a^
Se (μg/L)	606 ± 9.0^a^	599 ± 18^a^	566 ± 6.7^b^	606 ± 9.9^a^	583 ± 9.0^ab^

Each value is the mean ± SEM, n = 8. Values without a common letter differ, P < 0.05.

TCHO, total cholesterol; TG, triacylglycerol; TSH, thyroid-stlmulating hormone; T3, Triiodothyronine; T4, thyroxine; Se, Selenium. See the legend of Table 1 for other abbreviations.

**Figure 2:**
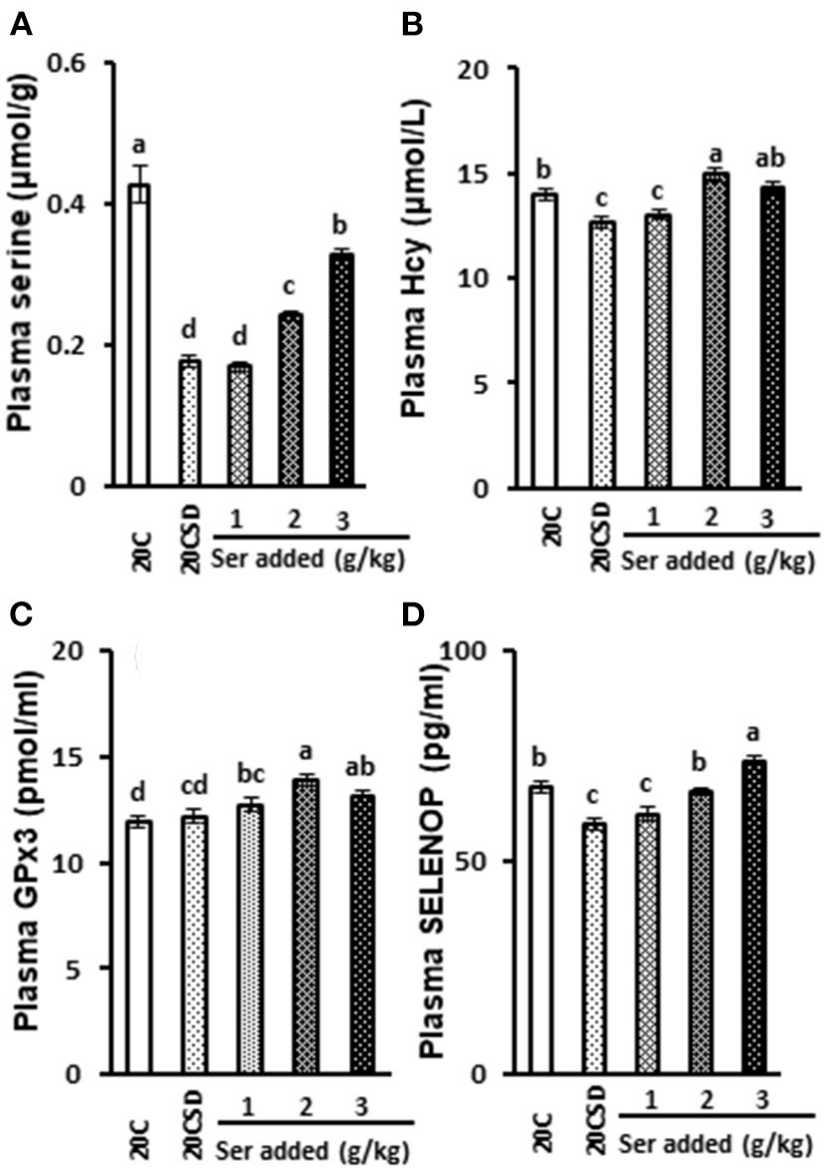
Effects of supplementation of serine-deprived diet with serine (0.5, 1.0, and 2.0 times the amount of serine in 20C) on plasma concentrations of serine **(A)**, Hcy **(B)**, GPx3 **(C)** and SELENOP **(D)** in rats fed 20% casein diets (experiment 1). Each value is the mean ± SEM, *n* = 8. Means in a panel without a common letter differ, *P* < 0.05. 1, 20% casein (20C); 2, serine-deprived 20C (20CSD); 3, 20CSD + 0.5 × Ser; 4, 20CSD + 1.0 × Ser; 5, 20CSD+2.0 × Ser.Ser, serine; Hcy, homocysteine; SELENOP, selenoprotein P; GPx3, Glutathione peroxidase 3.

Plasma serine concentration was significantly decreased in rats fed 20CSD compared with those a 20C (*P* < 0.05; Figure 2A). This serine deprivation-induced decrease was inhibited by serine supplementation in a dose-dependent manner (*P* < 0.05). Serine supplementation also significantly inhibited the serine deprivation-induced decrease of plasma homocysteine (*P* < 0.05; Figure 2B).

#### Changes in plasma selenoproteins

Plasma GPx3 concentration was not affected by any of the serine-deficient diets, but was increased by serine supplementation (*P* < 0.05; Figure 2C). SELENOP expression increased in a dose dependent manner with increasing doses of serine (*P* < 0.05; Figure 2D).

#### Pathological changes in the liver, kidney and pancreas

**Figure 3:**
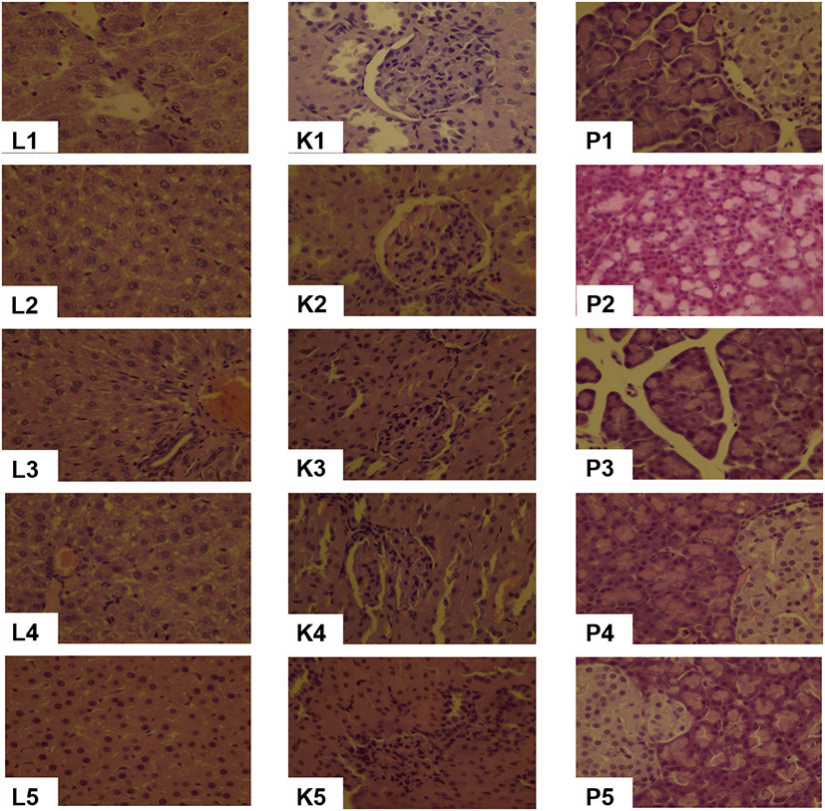
Effects of supplementation of serine-deprived diet with serine (0.5, 1.0, and 2.0 times the amount of serine in 20C) on Liver (L1–L5), Kidney (K1–K5) and Pancreas (P1–P5) pathological changes (10 × ) in rats fed 20C (experiment 1). 1, 20C; 2, 20CSD; 3, 20CSD + 0.5 × Ser; 4, 20CSD + 1.0 × Ser; 5, 20CSD + 2.0 × Ser.

In all diet groups, the liver and kidney tissues were normal and could be clearly identified whereas liver lobules, glomerular and tubular structure were all neatly arranged (Figure 3). Punctate necrosis of hepatocytes was observed in 20CSD and 20CSD + Ser groups. Significant glomerular atrophy was observed in the 20CSD group. Vacuole formation of varying sizes were seen in pancreatic epithelial cells of rats in all groups, indicating vacuolar degeneration in the pancreatic tissue. No significant pathological changes were observed in other organs such as the testes, brain, cerebellum, brainstem and femur (figure not listed).

#### GPx1 expression in the liver, kidney, brain and testes by immunohistochemical assay

**Figure 4:**
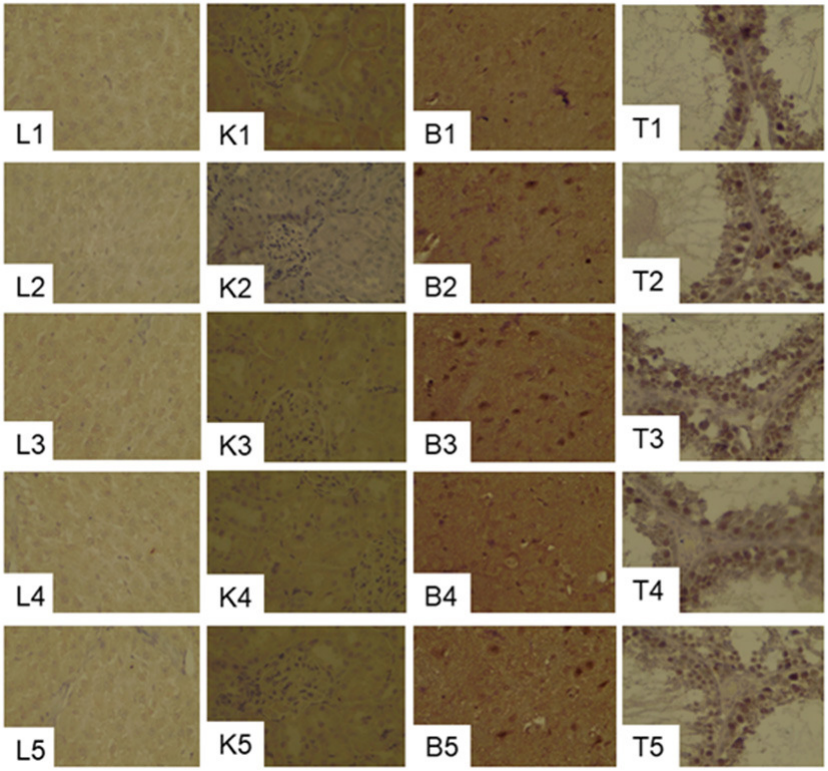
Effects of supplementation of serine-deprived diet with serine (0.5, 1.0, and 2.0 times the amount of serine in 20C) on the expression of GPx1in the liver (L1–L5), kidney (K1–K5), brain (B1–B5) and Testis (T1–T5) tissues in rats fed 20C (experiment 1). See the legend of Figure 3 for other abbreviations.

GPx1 gray scale in both liver and kidney were significantly decreased in the 20CSD group (P < 0.05). There were no significant differences in the GPx1 gray scale in other organs for all diet groups (Table 3 and Figure 4). It was shown that serine supplementation inhibited serine deprivation-induced decrease of GPx1 gray scale in the liver and kidney.

**Table 3:** Effects of supplementation of serine-deprived diet with serine (0.5, 1.0, and 2.0 times the amount of serine in 20C) on GPx1 gray scale at organs in rats fed a 20% casein diet (experiment 1).

**Diet**	**Liver**	**Kidney**	**Brian**	**Testis**
20C	142.4 ± 17.3^a^	165.7 ± 21.8^a^	133.6 ± 16.5	153.6 ± 21.7
20CSD	87.7 ± 21.5^b^	121.3 ± 21.7^b^	128.7 ± 23.7	161.8 ± 24.2
+ 0.5 Ser	135.4 ± 22.1^a^	157.9 ± 23.3^b^	132.4 ± 21.2	157.1 ± 20.2
+ 1.0 Ser	129.7 ± 21.7^a^	166.5 ± 21.5^a^	124.5 ± 24.1	142.3 ± 28.4
+ 2.0 Ser	134.5 ± 18.9^a^	153.4 ± 23.6^b^	138.1 ± 22.3	159.4 ± 23.6

Each value is the mean ± SEM, n = 8. Values without a common letter differ, P < 0.05. See the legend of Table 1 for other abbreviations.

### Experiment 2 — Conducted in pregnant rats and their offspring

#### Effects of PHGDH inhibitor and serine deficiency on plasma serine, Se, biochemical indicators and thyroid hormone

**Figure 5:**
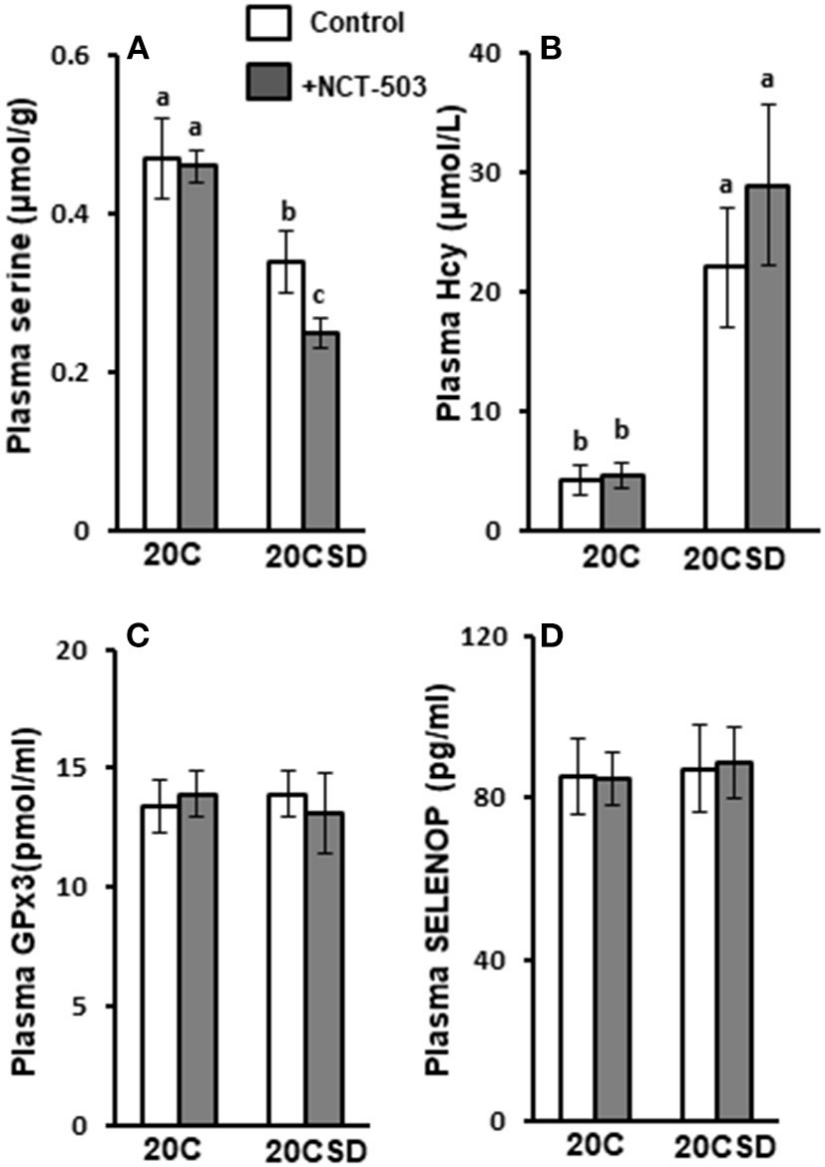
Effects of PHGDH inhibitor and serine deficiency on plasma concentrations of serine **(A)**, Hcy **(B)**, GPx3 **(C)** and SELENOP **(D)** in pregnant rats fed 20% casein (experiment 2). Each value is the mean ± SEM, *n* = 10. Means in a panel without a common letter differ, *P* < 0.05. See the legend of Figure 2 for other abbreviations. PHGDH, 3-phosphoglycerate dehydrogenase.

No differences were observed in blood TCHO, TG, TSH, T3 and T4 of pregnant rats assigned to all four diet groups (20C, 20C + NCT-503, 20CSD, 20CSD + NCT-503), indicating that these parameters were affected by neither exogenous serine deprivation nor endogenous serine synthesis dysfunction (Table 4). Plasma serine levels in 20CSD and 20CSD + NCT-503 were significantly lower than those in 20C and 20C + NCT-503 (*P* < 0.05; Figure 5A). Plasma Hcy was significantly higher in pregnant rats fed 20CSD (22.1 ± 7.8 μmol/L) compared to those in 20C (4.31 ± 1.28μmol/L; *P* < 0.05). On the other hand, plasma Hcy were not affected by PHGDH inhibitor regardless of serine adequacy in the diet of pregnant rats (Figure 5B).

**Table 4:** Effects of PHGDH inhibitor and serine deficiency on plasma TCHO concentration and other variables in pregnant rats fed 20% casein diets (experiment 2).

	**20C**	**20C + PHGDH**	**20CSD**	**20CSD + PHGDH**
TCHO (mmol/L)	2.65 ± 0.33	2.59 ± 0.39	2.53 ± 0.63	2.42 ± 0.67
TG (mmol/L)	0.27 ± 0.09	0.28 ± 0.06	0.29 ± 0.31	0.18 ± 0.16
TSH (μIU/L)	1,066 ± 94	1,098 ± 104	1,026 ± 105	1,080 ± 124
T3 (pmol/L)	38.0 ± 3.0	38.2 ± 3.6	37.5 ± 4.2	39.8 ± 4.3
T4 (pmol/L)	1,484 ± 192	1,455 ± 158	1,506 ± 145	1,478 ± 176
Se (μg/L)	407 ± 21	457 ± 35	598 ± 49	588 ± 59

Each value is the mean ± SEM, n = 10. See the legend of Table 1 for other abbreviations.

#### Effects of PHGDH inhibitor and serine deficiency on plasma concentrations of GPx3 and SELENOP

Plasma GPx3 and SELENOP concentrations were similar in all groups regardless of serine status (Figures 5C,D).

#### Effects of PHGDH inhibitor and serine deficiency on DIO1 and DIO2 expression

**Figure 6:**
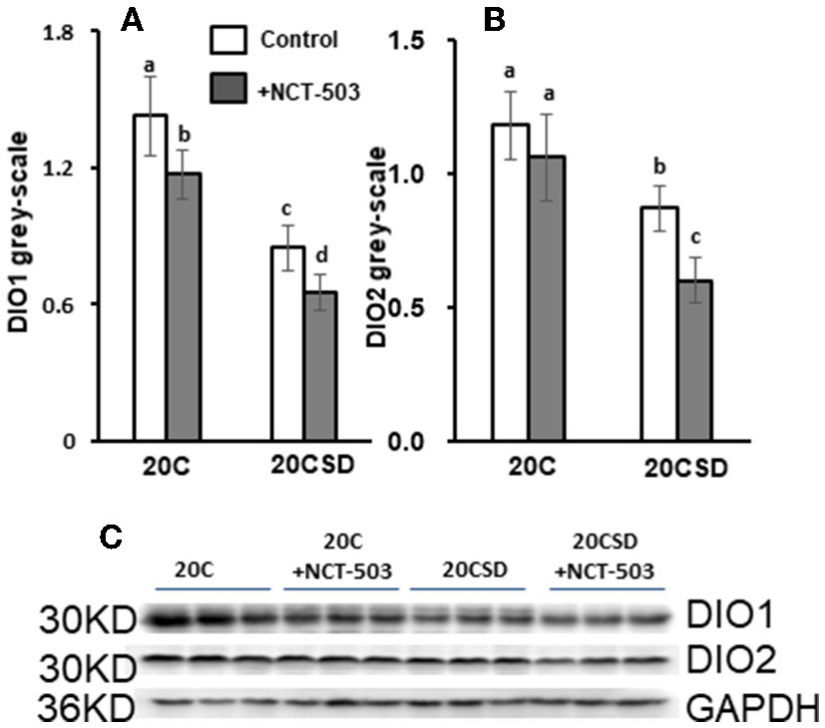
Effects of PHGDH inhibitor and serine deficiency on DIO1 and DIO2 expression in pregnant rats fed 20% casein (experiment 2). Each value is the mean ± SEM. Means in a panel without a common letter differ, *P* < 0.05. DIO1, iodothyronine deiodinase 1; DIO2, iodothyronine deiodinase 2; GAPDH, glyceraldehyde-3-phosphate dehydrogenase.

Compared to 20C, gray-scale of DIO1 and DIO2 were significantly lower in serine-deficient rats (*P* < 0.05; Figures 6A–C). PHGDH further exacerbated the decrease of DIO1 and DIO2 in rats fed 20CSD. The above indicated that serine deprivation and PHGDH inhibitor reduce the expression of DIO1 and DIO2 in the liver.

#### Effects of PHGDH inhibitor on GPx1 expression in the liver, kidney, brain and testes

**Figure 7:**
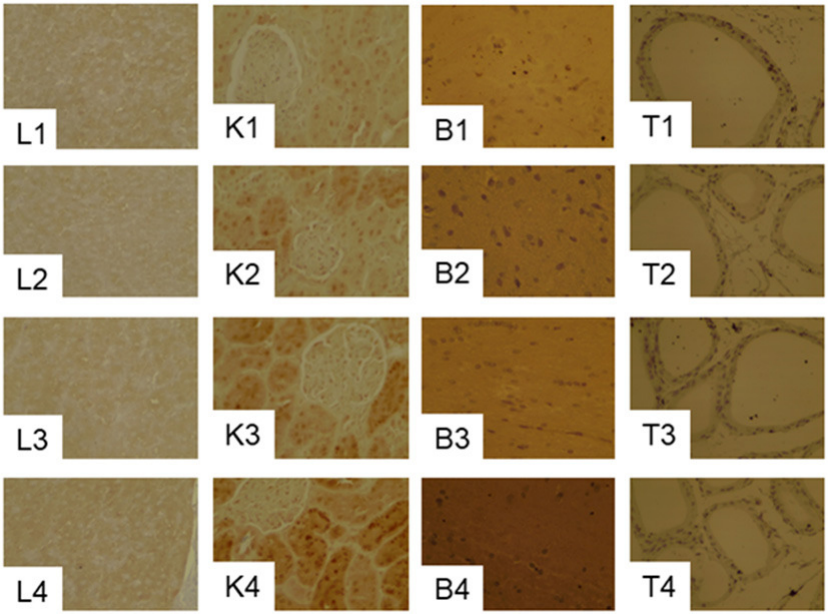
Effects of PHGDH inhibitor and serine deficiency on the expression of GPx1in the liver (L1–L4), kidney (K1–K4), brain (B1–B4) and Testis (T1–T4) tissues in pregnant rats fed 20% casein (experiment 2). See the legend of Figure 7 for other abbreviations.

GPx1 gray scale in the liver and kidney were markedly lower in the 20CSD group compared to the 20C group (*P* < 0.05); and were unaffected by the presence of PHGDH inhibitor (Table 5 and Figure 7). The above indicated that serine deprivation only reduced GPx1 expression in the liver and kidney but not in other organs such as thyroid and brain.

**Table 5:** Effects of PHGDH inhibitor and serine deficiency on GPx1 gray scale at organs in pregnant rats fed a 20% casein diets (experiment 2).

**Diet**	**Liver**	**Kidney**	**Brian**	**Thyroid**
20C	153.5 ± 18.4^a^	146.0 ± 21.1^a^	167.5 ± 25.4	134.6 ± 23.6
20C+PHGDH	141.1 ± 19.7^a^	152.6 ± 24.2^a^	164.8 ± 23.3	141.3 ± 25.4
20CSD	92.9 ± 17.8^b^	89.4 ± 14.7^b^	162.6 ± 27.8	136.2 ± 20.4
20CSD + PHGDH	98.2 ± 13.5^b^	90.1 ± 16.2^b^	155.3 ± 23.5	125.9 ± 25.5

Each value is the mean ± SEM, n = 10. Values without a common letter differ, P < 0.05. See the legend of Table 1 for other abbreviations.

#### Pathological changes in the liver, kidney and pancreas

**Figure 8:**
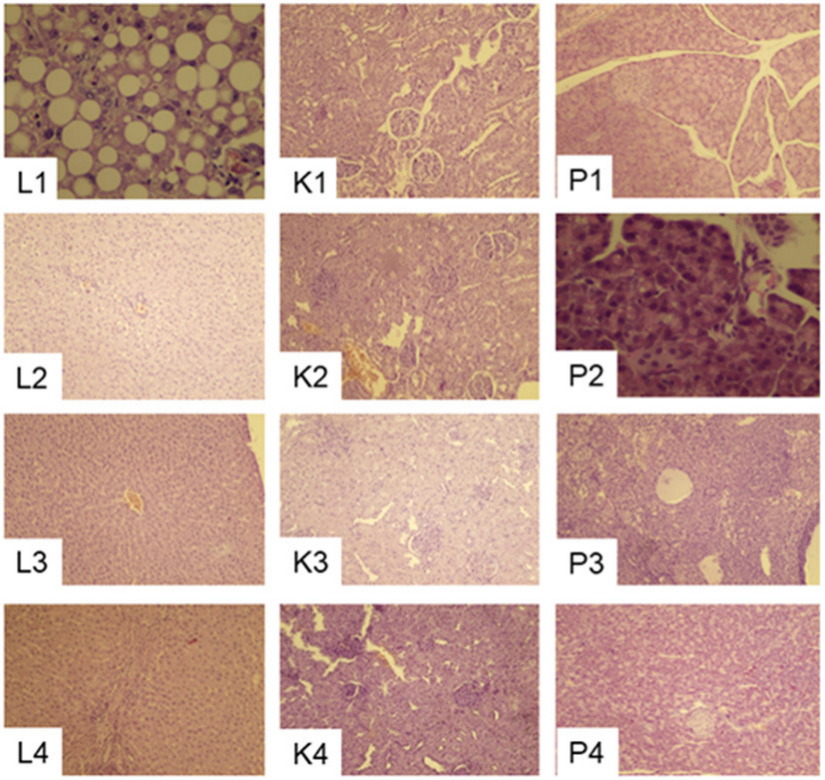
Effects of PHGDH inhibitor and serine deficiency on Liver (L1–L4), Kidney (K1–K4) and Pancreas (P1–P4) pathological changes (10 × ) in pregnant rats fed 20% casein (experiment 2). 1, 20C; 2, 20C+NCT-503; 3, 20CSD; 4, 20CSD + NCT-503.

Pregnant rats fed 20C had Liver tissues with normal structures that can be clearly identified, liver lobules were neatly arranged, and the hepatocytes were organized radially around the central vein (Figure 8). Hydropic degeneration of the liver, punctate necrosis of hepatocytes and inflammatory cell infiltration in the confluent area were observed in those fed 20CSD and 20CSD + NCT-503. Significant glomerular atrophy was observed in these groups. Vacuole formation of varying sizes were also seen in pancreatic epithelial cells of rats fed 20CSD and 20CSD + NCT-503, indicating vacuolar degenerative changes in pancreatic tissue. No significant pathological changes were observed in other organs including the thyroid, ovary, uterus, brain, cerebellum and brain stem for all diet groups (figure not listed).

#### Physiologic development and health of offspring born to pregnant rats with endogenous and exogenous serine deficiency

**Figure 9:**
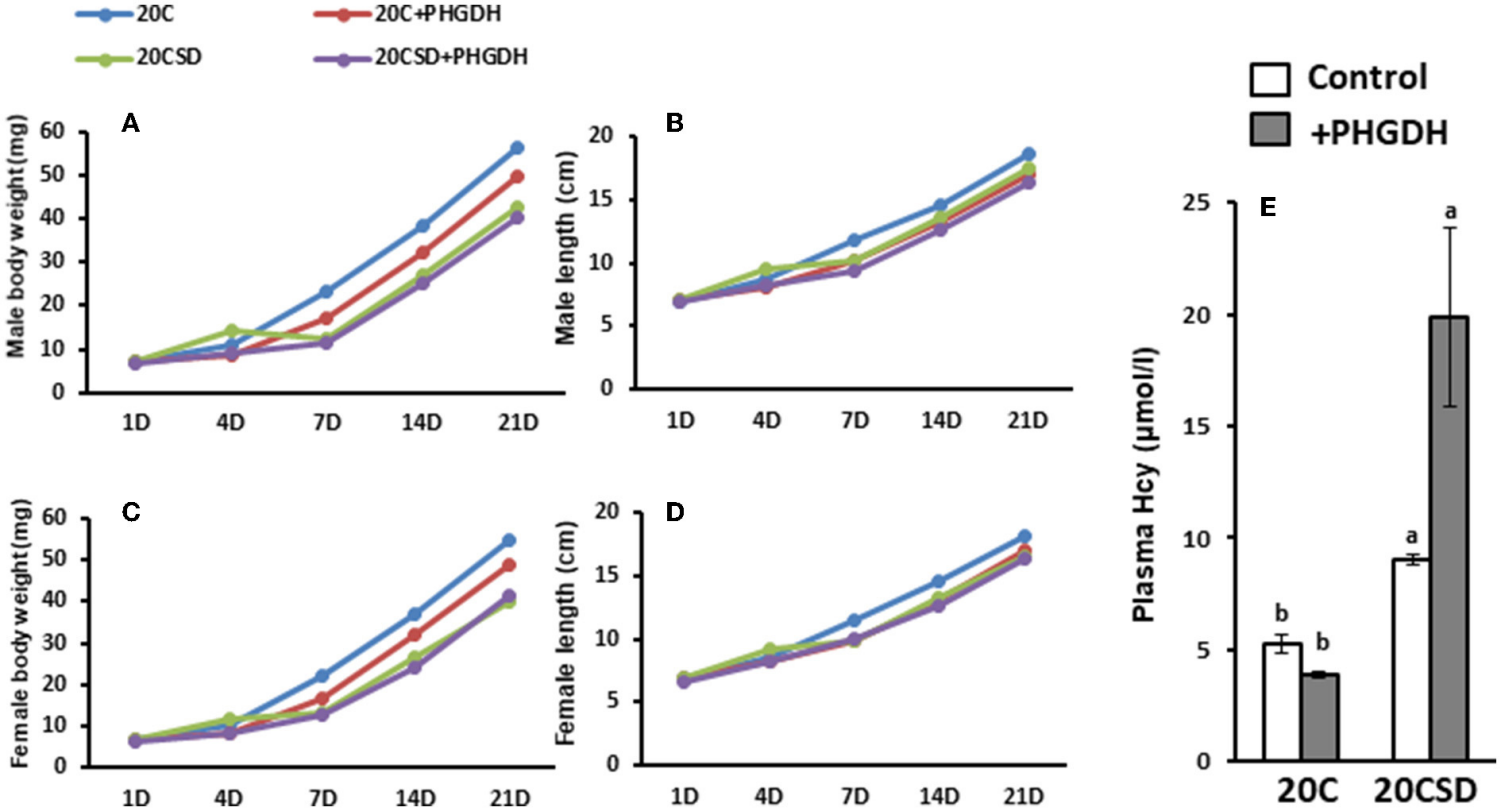
Effects of Low plasma serine and normal selenoprotein concentration in pregnant rats on the physiologic development and health of offsprings (experiment 2). Each value is the mean ± SEM, *n* = 8. Means in a panel without a common letter differ, *P* < 0.05. See the legend of Figure 5 for other abbreviations.

Although there were no significant differences in offspring body weight and length from birth to day 21, it was observed that offspring from mothers fed a serine sufficient diet (20C and 20C + NCT-503) had tended to be higher body weight compared to those from mothers fed a serine deficient diet (20CSD and 20CSD + NCT-503; Figures 9A–D). Hcy concentration was significantly elevated in maternal rats fed 20CSD compared with those fed 20C (*P* < 0.05; Figure 9E). Furthermore, hyperhomocysteinemia were found in offspring of pregnant rats administrated with PHGDH inhibitor.

## Discussion

Although serine is a nonessential amino acid in terms of nutrition, it is vital for the normal physiological function of selenium due to its direct or indirect participation in selenium metabolism and selenoprotein synthesis (18, 19). Furthermore, serine is a key component in the de novo synthesis of selenocysteine (20, 21).

To date, most studies (3, 22) showed serine supplementation increased selenoprotein expression or enzyme activity but few reported the impact of insufficient exogenous and/or endogenous serine on selenoprotein expression and their biological functions in cells.

This study consisted of two experiments. Firstly, a gradient model of exogenous serine deficiency was established by supplementing different concentrations of serine in male rats fed with a serine deficient diet. This was then combined with an anti-tumor model (23) (PHGDH enzyme inhibition animal model) to set up a novel exogenous and/or endogenous serine deficient pregnant rat model.

**Figure 10:**
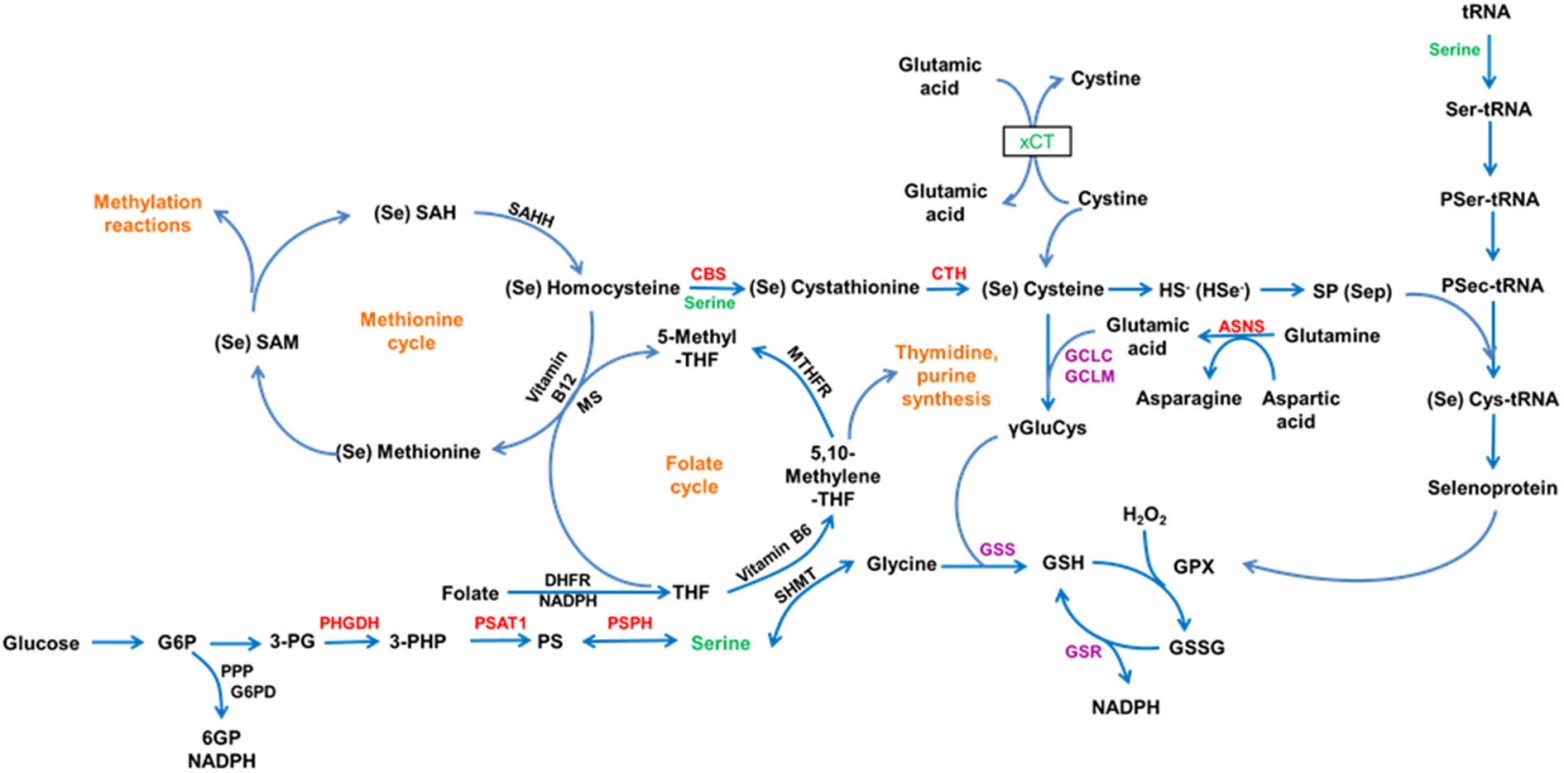
This schematic illustrates the role of Serine in selenium metabolism and function.

In Experiment 1, plasma serine concentration of male rats increased with increasing serine concentration in a serine deficient diet. Dietary serine concentration was positively associated with plasma selenoprotein including selenoprotein P (SELENOP) and GPx3 concentration (4). This may be due to the fact that serine plays a pivotal role in the synthesis of selenocysteine (Figure 10), and exogenous serine can affect the synthesis of selenoproteins in the body. It is known that the biological activity of thyroid hormone T3 is much higher than that of T4; plasma T3 is transformed from T4 in tissues by deiodinases, a Se-containing enzyme (24). Plasma T4 increased with increasing serine concentration in a serine deficient diet; this was not observed in the case of plasma T3. Further research is necessary to determine the effects of dietary serine on deiodinase levels in organs and tissues.

Experiment 1 demonstrated that serine deprivation decreased TG levels in male rats but was inhibited by serine supplementation. Glycine and serine play different roles in the metabolism of methionine; glycine acts as a methyl-group acceptor whereas serine is a substrate for cystathionine systhesis. Glycine and serine can be mutually converted in a reaction catalyzed by serine hydroxymethyltransferase, but the reaction is known to favor serine synthesis (25). Phosphatidylcholine (PC) is synthesized via either the CDP-choline pathway or phosphatidylethanolamine (PE) *N*-methylation pathway (26, 27). Although s-adenosyl methionine (SAM), PC and PE *N*-methyltransferase levels were not investigated in this study, it is presumed that the increase in PC synthesis by serine supplementation resulted from either the decrease of hepatic SAM concentration in the PE *N*-methylation pathway or by competition between PE-*N* methyltransferase and serine hydroxymethyltransferase. This increase in PC concentration resulted in high TG levels in serine deprived male rats fed serine supplementation. On the other hand, serine is involved in the regulation of hepatic lipid metabolism. He (28) reported exogenous and endogenous serine deficiencies exacerbated hepatic lipid accumulation via inhibition of serine de novo biosynthesis resulted in hepatic serine deficiency and further contributed to the fatty liver. And dietary serine deficiency altered the microbiota composition and further resulted in the development of fatty liver via the gut-microbiota-liver axis.

In Experiment 2, plasma serine concentration in pregnant rats was associated with dietary serine concentration but selenoprotein (including SELENOP and GPx3) levels were not affected. The same was also observed in pregnant rats given a PHGDH inhibitor (NCT-503), where plasma serine concentration decreased but plasma selenoproteins were not affected. Drastic change in DIO1/2 expression was found in liver of pregnant rats on serine-deficent diet (20CSD) or with impaired endogenous serine synthesis (20C + NCT-503; 20CSD + NCT-503). A possible explanation is that pregnant rats may have an adaptive mechanism to limit maternal serine utilization and ensure adequate supply to the fetus when the supply of serine is insufficient (caused by either insufficient exogenous serine or impaired endogenous serine synthesis) (27, 29). This was supported by a decrease in selenoprotein (DIO1/2) synthesis in the maternal organs and tissues but an increase in plasma selenoproteins (SELENOP and GPx3) concentration for transport to the fetus (30). Furthermore, low expression of liver DIO1/2 did not led to changes in plasma T4 and T3 in pregnant rats either on a low serine diet or given NCT503.

What is interesting about the present study is that serine deprivation significantly increased plasma Hcy concentration in pregnant rats, whilst the opposite was observed in male rats. However, the decrease in plasma Hcy in male rats was significantly inhibited by serine supplementation in a dose-dependent manner. Serine suppressed hyperhomocysteinemia in rats can be induced by choline deficiency or methionine (31, 32). One may wonder why plasma Hcy concentrations differ in pregnant rats and male rats when subjected to the same serine deficient dietary. Hypohomocysteinemic was found in pregnant rats fed 20C diet; serine deprivation increased plasma Hcy concentration but were still below the normal Hcy level. Protein requirements are higher in pregnant rats compared to those not pregnant (33). The possible mechanism is due to the preferential involvement of endogenous serine in selenoproteins synthesis when exogenous serine is absent. Endogenous serine is produced not only by the glycolysis pathway, but also from the transformation of glycine. On the other hand, endogenously synthesized serine acts as a methyl donor for 5-MTHF in the folic acid cycle for the synthesis of SAM, and also for the methylation of excess selenium (33). Our previous result showed serine played a direct role in selenoprotein expression through the *de novo* biosynthesis of Sec-tRNA^[Ser]Sec^ *in vitro* (2); this was also supported *in vivo* by other studies (34, 35). Dietary serine and sulfur-containing AAs were found to have a direct effect on the nutritional status of lactating Chinese women (5); thus a high protein intake or low plasma SEPP1 may pose as health risks in this population. TG and TCHO of male rats were decreased due to serine deprivation, but there were no changes in the pregnant mice in Experiment 2. Serine is closely related to lipid metabolism as serine and fatty acid forms ceramide, and in turn phospholipids. Thus we were interested in looking at the effects of serine deprivation on blood lipid (profile). To our current knowledge and findings, we can only speculate that the lack of changes in the lipid profile observed in pregnant rats were due to either (a) influence of pregnancy hormones or (b) other biological mechanisms during serine deficiency. We will definitely look into this in future studies.

As expected, plasma homocysteine level of offspring were consistent with that of their mothers; serine deficiency in the mothers were reflected by hyperhomocysteinemia in their offsprings. Growth and development of offspring were also stunted by the lack of maternal serine.

In a nutshell, endogenous or exogenous serine deficiency reduced selenoprotein synthesis in rats. However, this was not observed in pregnant rats, we assume that this is to ensure adequate selenium supply to the offspring. Reflected by pathological changes in the liver and kidney, insufficient maternal serine had negative effects on the health of pregnant rats; hyperhomocysteinemia and growth retardation were also found in offspring born to pregnant rats with insufficient serine. Further studies are needed to observe whether the adverse outcomes observed in the offspring can be gradually improved after weaning via a diet containing an optimal amount of serine.

## Data availability statement

The original contributions presented in the study are included in the article/supplementary material, further inquiries can be directed to the corresponding authors.

## Ethics statement

The animal study was reviewed and approved by the Animal Care and Use Committee of National Institute of Nutrition and Health, Chinese Centre for Disease Prevention and Control.

## Author contributions

YL and JW contributed equally in this work, responsible for animal experiments. QW and FH provided valuable advice on the writing. LS and CH participated in the WB experiment. ZH and LX designed the experiments and revised the article critically for important content. All authors approved the final manuscript.

## Funding

The authors would like to thank the financial support of the National Key Research and Development Program of China (grant number 2020YFC2006302), the National Natural Science Foundation of China under Grant Nos. 81741032 and 81770745.

## Conflict of interest

The authors declare that the research was conducted in the absence of any commercial or financial relationships that could be construed as a potential conflict of interest.

## Publisher's note

All claims expressed in this article are solely those of the authors and do not necessarily represent those of their affiliated organizations, or those of the publisher, the editors and the reviewers. Any product that may be evaluated in this article, or claim that may be made by its manufacturer, is not guaranteed or endorsed by the publisher.
